# Glycemic Variability Is an Independent Predictive Factor for Development of Hepatic Fibrosis in Nonalcoholic Fatty Liver Disease

**DOI:** 10.1371/journal.pone.0076161

**Published:** 2013-11-06

**Authors:** Motoi Hashiba, Masafumi Ono, Hideyuki Hyogo, Yukio Ikeda, Kosei Masuda, Reiko Yoshioka, Yoichi Ishikawa, Yuri Nagata, Kensuke Munekage, Tsunehiro Ochi, Akira Hirose, Yasuko Nozaki-Fujimura, Shuhei Noguchi, Nobuto Okamoto, Kazuaki Chayama, Narufumi Suganuma, Toshiji Saibara

**Affiliations:** 1 Department of Gastroenterology and Hepatology, Kochi Medical School, Kochi, Japan; 2 Department of Medicine and Molecular Science, Graduate School of Biomedical Sciences, Hiroshima University, Hiroshima, Japan; 3 Diabetes Center, Kochi Memorial Hospital, Kochi, Japan; 4 Department of Environmental Medicine, Kochi Medical School, Kochi, Japan; University College London, United Kingdom

## Abstract

Patients with nonalcoholic fatty liver disease (NAFLD) and nonalcoholic steatohepatitis (NASH) often have metabolic disorders including insulin resistance and type 2 diabetes mellitus (T2DM). We clarified the predictive factors in glucose metabolism for progression of hepatic fibrosis in patients with NAFLD by the 75-g oral glucose tolerance test (75gOGTT) and a continuous glucose monitoring system (CGMS). One hundred sixty-nine patients (68 female and 101 male patients) with biopsy-proven NAFLD with performance with 75gOGTT were enrolled and divided into four groups according to the stage of hepatic fibrosis (F0–3). The proportion of patients with T2DM significantly gradually increased, HbA1c and the homeostasis model assessment of insulin resistance were significantly elevated, and 1,5-anhydroglucitol (1,5-AG) was remarkably decreased with the progression of fibrosis. In the 75gOGTT, both plasma glucose and insulin secretion were remarkably increased with the progression of fibrosis. The only factor significantly associated with advanced fibrosis was 1,5-AG (*P* = 0.008) as determined by multivariate logistic regression analysis. We next evaluated the changes in blood glucose during 24 hours by monitoring with the CGMS to confirm the relationship between glycemic variability and progression of fibrosis. Variability of median glucose, standard deviation of median glucose (*P* = 0.0022), maximum blood glucose (*P* = 0.0019), and ΔMin–max blood glucose (*P* = 0.0029) were remarkably higher in severe fibrosis than in mild fibrosis.

**Conclusion:**

Hyperinsulinemia and hyperglycemia, especially glycemic variability, are important predictive factors in glucose impairment for the progression of hepatic fibrosis in NAFLD.

## Introduction

Nonalcoholic fatty liver disease (NAFLD) includes a wide spectrum of liver diseases that range from simple steatosis, which is usually a benign and non-progressive condition, to nonalcoholic steatohepatitis (NASH), which can progress to liver cirrhosis (LC) and hepatocellular carcinoma in the absence of significant alcohol consumption [1]–[3]. The progression of hepatic fibrosis is an important predictive factor for the development of LC and hepatocellular carcinoma not only in patients with chronic hepatitis C, but also in those with NASH [4]. To inhibit the progression of hepatic fibrosis in NASH, it is important to clarify the predictive factors for progression of hepatic fibrosis.

NASH and NAFLD are considered to be hepatic manifestations of the metabolic syndrome including insulin resistance (IR) and abnormalities of glucose metabolism [5], [6]. In accordance with the increased prevalence of obesity and type 2 diabetes mellitus (T2DM) in the general population worldwide, the number of patients with NASH and NAFLD have also increased [7], [8]. T2DM is considered to be an independent risk factor for the development of NASH and NAFLD [9], [10], and hyperinsulinemia and hyperglycemia are common not only in obese patients, but also in non-obese, non-diabetic patients with NASH [11]. On the other hand, the presence of NASH and NAFLD themselves is also considered to be associated with a high risk of developing T2DM [12].

Postprandial hyperglycemia and glycemic variability were reported to involve progression of atherosclerosis through increase of oxidative stress, activation of inflammatory cytokines and inflammation [13]–[15]. Oxidative stress is well known as one of most important factor for inflammation and progression of hepatic fibrosis in NAFLD patients [16], [17]. The continuous glucose monitoring system (CGMS) has been introduced as a useful tool, which detect postprandial hyperglycemia [18] and glycemic variability during 24 hours in DM patients. In addition, episodic hypoglycemia during sleeping time can also be detected by CGMS [19]. However, postprandial hyperglycemia and glycemic variability have not yet been evaluated by CGMS in NAFLD patients. Moreover, the relationship between the clinical features of glucose impairment and the progression of hepatic fibrosis in NASH and NAFLD has not been well elucidated. In this study, therefore, we clarified the predictive factors in glucose metabolism for the progression of hepatic fibrosis in NAFLD using the 75-g oral glucose tolerance test (75gOGTT) and CGMS.

## Patients and Methods

### Patients

A total of 169 patients with biopsy-proven NAFLD (68 female and 101 male patients) with performance with 75gOGTT were enrolled in this study. Liver biopsies had been obtained in all patients after a thorough clinical evaluation had been performed and signed informed consent had been obtained from each patient. Patients with known use of methotrexate, tamoxifen, corticosteroids, or alcohol in excess of 20 g per day and patients with other known causes of liver disease including viral hepatitis, hemochromatosis, Wilson's disease, and autoimmune liver diseases were excluded from this study. None of the patients had received anti-diabetic drugs or insulin. The study protocol conformed to the ethical guidelines of the 1975 Declaration of Helsinki [20] and was approved by the Research Committee of Kochi Medical School.

### Clinical and Laboratory Evaluation

Venous blood samples were obtained in the morning after a 12-hour overnight fast. Laboratory tests in all patients included measurements of serum aspartate aminotransferase, alanine aminotransferase, gamma-glutamyl transpeptidase, lipid profiles, total cholesterol, triglycerides, high-density lipoprotein cholesterol, low-density lipoprotein cholesterol, fasting plasma glucose, fasting immunoreactive insulin (f-IRI), creatinine, blood urea nitrogen, 1,5-anhydroglucitol (1,5-AG), HbA1c, and fibrosis markers. These parameters were measured using standard clinical chemistry techniques in the laboratory section of Kochi Medical School Hospital. All patients underwent the 75gOGTT. Plasma glucose and insulin concentrations were measured at 0, 30, 60, 90, 120, and 180 minutes. Insulin resistance was calculated by the homeostasis model (HOMA)-IR using following formula: HOMA-IR =  fasting plasma insulin (μU/ml) X fasting plasma glucose (mg/dl)/405. The measure of insulin secretion was calculated by the insulinogenic index using following formula: insulinogenic index  =  (Δplasma insulin 0–30 min)/(Δplasma glucose 0–30 min).

### Histological Evaluation

Liver biopsies of all patients were performed percutaneously under ultrasonographic guidance, and biopsy specimens were obtained from the liver parenchyma of the upper region of the right lobe using a 15-gauge biopsy needle. Liver biopsy specimens were routinely fixed in 10% phosphate-buffered formalin (pH 7.4), embedded in paraffin, and sectioned for hematoxylin and eosin staining. Hepatic fibrosis was assessed by Brunt's classification [21], and fibrosis staging was as follows: 0 =  no fibrosis; 1 =  zone 3 fibrosis only; 2 =  zone 3 and portal/periportal fibrosis; 3 =  bridging fibrosis; and 4 =  cirrhosis. Histological evaluation was performed by two pathologists with no knowledge of the patients' clinical data.

### Continuous Glucose Monitoring System (CGMS)

Continuous glucose levels in 20 patients with biopsy-proven NAFLD were monitored by the CGMS System Gold (Medtronic MiniMed, Northridge, CA, USA). None of the patients had received anti-diabetic drugs, including insulin injection. In the severe hepatic fibrosis group, four patients with NAFLD with F4 fibrosis (LC) were included in this study, unlike in the 75gOGTT study. According to the operating guidelines, the CGMS was installed in the patients to monitor the glucose levels of interstitial fluid [22]. The glucose sensor was inserted into the subcutaneous tissue of the abdomen at 3∶00 to 4∶00 PM and was monitored for 30 hours. Finger-stick blood glucose levels were checked to calibrate the first glucose value of the CGMS after 1 hour of initialization. Glucose concentrations were determined at least four times per day with an automatic blood glucose meter (Glutest; Sanwa Kagaku Kenkyusho Co., Ltd., Nagoya, Japan). Meals were strictly standardized (1800 kcal/day of standard diets at Kochi Medical School Hospital) during the examination.

### Statistical Analyses

Results are presented as mean ± standard deviation for quantitative data and as numbers or percentages for categorical or qualitative data. Statistical differences in quantitative data were determined using the Mann-Whitney U test or post-hoc test. Qualitative data were compared using the chi-square test. Multivariate analysis was carried out by logistic regression. These statistical analyses were carried out using Small Stata 10.1 for Windows. Results were considered significant when the P value was <0.05.

## Results

### Relationship between glucose impairment and progression of hepatic fibrosis

**Figure 1:**
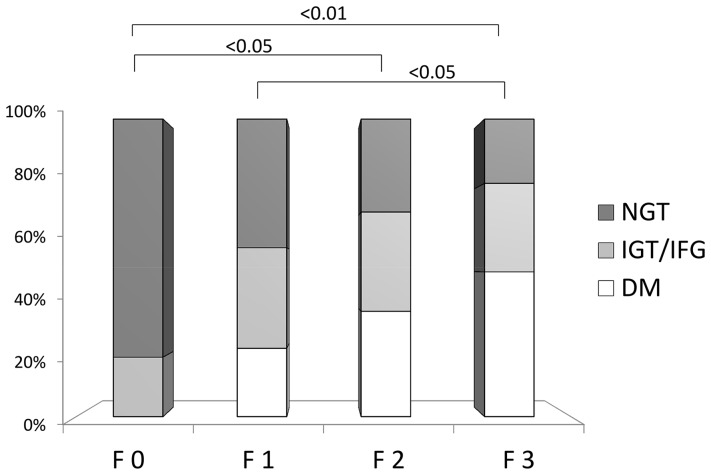
Relationship between glucose impairment and progression of hepatic fibrosis. The frequencies of NGT, IGT/IFG, and T2DM in the four stages of hepatic fibrosis are shown. The diagnosis of glucose impairment was based on the 75gOGTT. The prevalence of NGT in patients in the F0 group (80.0%) was significantly higher than that in the F1 (43.2%), F2 (31.3%), and F3 groups (21.6%), and the frequencies of patients with T2DM in the F3 group (48.6%) was significantly higher than that in the F0 (0%), F1 (22.9%), and F2 (35.4%) groups. *P*-values were calculated using the X^2^-test. Fibrosis stage (F): F0 (*n* = 10), F1 (*n* = 74), F2 (*n* = 48), F3 (*n* = 37); total, N = 169.

To investigate the relationship among clinical features of glucose levels, insulin secretion, and hepatic fibrosis, the 169 patients with NAFLD were classified into four groups based on the stage of hepatic fibrosis stage: F0 (*n* = 10), F1 (*n* = 74), F2 (*n* = 48), and F3 (*n* = 37). The clinical and physiological data of the four groups are shown in Table 1. The hepatic fibrosis markers hyaluronic acid, type IV collagen 7S, and type III procollagen N-peptide were significantly increased according to the progression of hepatic fibrosis. In addition, the patients with severe fibrosis were much older and had higher ferritin and transaminase levels. The platelet count tended to decrease according to the progression of hepatic fibrosis. We next evaluated the relationship between the prevalence of T2DM diagnosed by 75gOGTT and the stage of hepatic fibrosis in patients with NAFLD. The prevalence of patients with normal glucose tolerance (NGT) was 80%, and no patients with T2DM were found in the F0 group (Fig. 1). On the other hand, the prevalence of NGT in the patients with F3 disease was only 21.6%, and the prevalence of T2DM was 48.6%. In accordance with the progression of hepatic fibrosis, the prevalence of patients with T2DM was significantly gradually increased (F0 versus F2, *P*<0.05; F0 versus F3, *P*<0.01; F1 versus F3, *P*<0.05). To clarify the factors of glucose impairment that are related to the progression of hepatic fibrosis, we evaluated the various parameters of glucose metabolism.

**Table 1:** Clinical and physiological characteristics of patients with NAFLD in the four stages of hepatic fibrosis.

	F0 (n = 10)			F1 (n = 74)			F2 (n = 48)			F3 (n = 37)		
Gender (F/M)	4/6			25/49			18/30			21/16		
Age (yo)	39.6	±	12.2	45.6	±	15.4	49.1	±	14.2	53.6	±	14.3*** ^,##^
BMI (kg/m^2^)	27.4	±	3.0	27.6	±	5.5	28.3	±	4.3	30.1	±	4.6 ^##^
AST (IU/L)	37.8	±	11.1	43.4	±	20.9	54.5	±	32.8 ^#^	79.3	±	46.1**^,###, ++^
ALT (IU/L)	64.5	±	29.4	78.2	±	37.1	99.8	±	67.6 ^#^	111.1	±	68.1*^, #^
ALP (IU/L)	207.2	±	109.9	240.8	±	109.8	287.8	±	100.7	253.1	±	106.3
GGT (IU/L)	113.6	±	101.3	58.2	±	42.9 **	79.7	±	40.0	91.8	±	80.3 ^#^
ChE (IU/L)	375.9	±	59.2	359.8	±	77.7	371.5	±	54.8	328.0	±	82.6
Albumin (g/dl)	4.44	±	0.22	4.58	±	0.28	4.45	±	0.28	4.45	±	0.31
BUN (mg/dl)	13.8	±	2.6	13.8	±	4.0	14.8	±	4.9	13.7	±	3.9
Crn (mg/dl)	0.69	±	0.11	0.84	±	0.97	0.66	±	0.22	0.68	±	0.12
UA (mg/dl)	6.38	±	1.70	6.42	±	2.21	6.38	±	1.29	5.85	±	1.08
FPG (mg/dl)	90.8	±	8.6	104.6	±	24.1	100.6	±	15.5	106.5	±	20.9 *
HbA1c (%)	5.46	±	0.22	5.92	±	1.14	5.96	±	0.77 *	6.27	±	0.98 *
T-Cho (mg/dl)	214.6	±	39.8	203.5	±	35.3	222.2	±	29.8 ^#^	215.3	±	53.7 ^+^
TG (mg/dl)	185.9	±	144.9	150.4	±	90.6	186.8	±	98.0 ^#^	142.7	±	55.0 ^+^
RBC (×10^4^/ml)	467.0	±	49.5	478.9	±	49.0	446.4	±	38.9	445.1	±	38.2 ^#^
Hb (g/dl)	14.1	±	1.4	14.3	±	1.8	13.9	±	1.6	13.9	±	0.8
Plt (×10^4^/ml)	24.0	±	5.3	23.7	±	5.9	22.8	±	3.8 ^#^	20.0	±	5.4^ # #^
WBC (×10^3^/ml)	4.47	±	3.18	5.29	±	2.54	5.42	±	2.64	4.02	±	3.10
Fe (mg/dl)	93.2	±	17.6	106.4	±	30.6	107.2	±	20.6	127.1	±	42.6 ^#^
Ferritin (ng/ml)	155.7	±	131.0	241.7	±	172.9	262.9	±	192.6	328.3	±	277.2 ^#^
HA (ng/ml)	20.2	±	13.5	30.4	±	19.5	34.1	±	26.3	93.5	±	105.0 ^###, +++^
IVcollagen7S (ng/ml)	2.79	±	0.52	3.52	±	0.67 **	3.77	±	1.01 **	5.05	±	2.35*^,###, ++^
P-3-P (U/ml)	0.53	±	0.14	0.57	±	0.17	0.69	±	0.24	0.92	±	0.66 ^#^

P-values were calculated using the Mann–Whitney U test. Versus F0: *P<0.05, **P<0.01, ***P<0.001. Versus F1: ^#^P<0.05, ^##^P<0.01, ^###^P<0.001. Versus F2: ^++^P<0.01, ^+++^P<0.001. Fibrosis stage (F): F0 (n = 10), F1 (n = 74), F2 (n = 48), F3 (n = 37); total, N = 169. BMI, body mass index; AST, aspartate aminotransferase; ALT, alanine aminotransferase; GGT, gamma-glutamyl transpeptidase; ChE, cholinesterase; UA, uric acid; T-Cho, total cholesterol; TG, triglycerides; FPG, fasting plasma glucose; Plt, platelets; Fe, plasma iron; HA, hyaluronic acid; IV collagen 7S, type IV collagen 7S; P-3-P, type III procollagen N-peptide.

**Figure 2:**
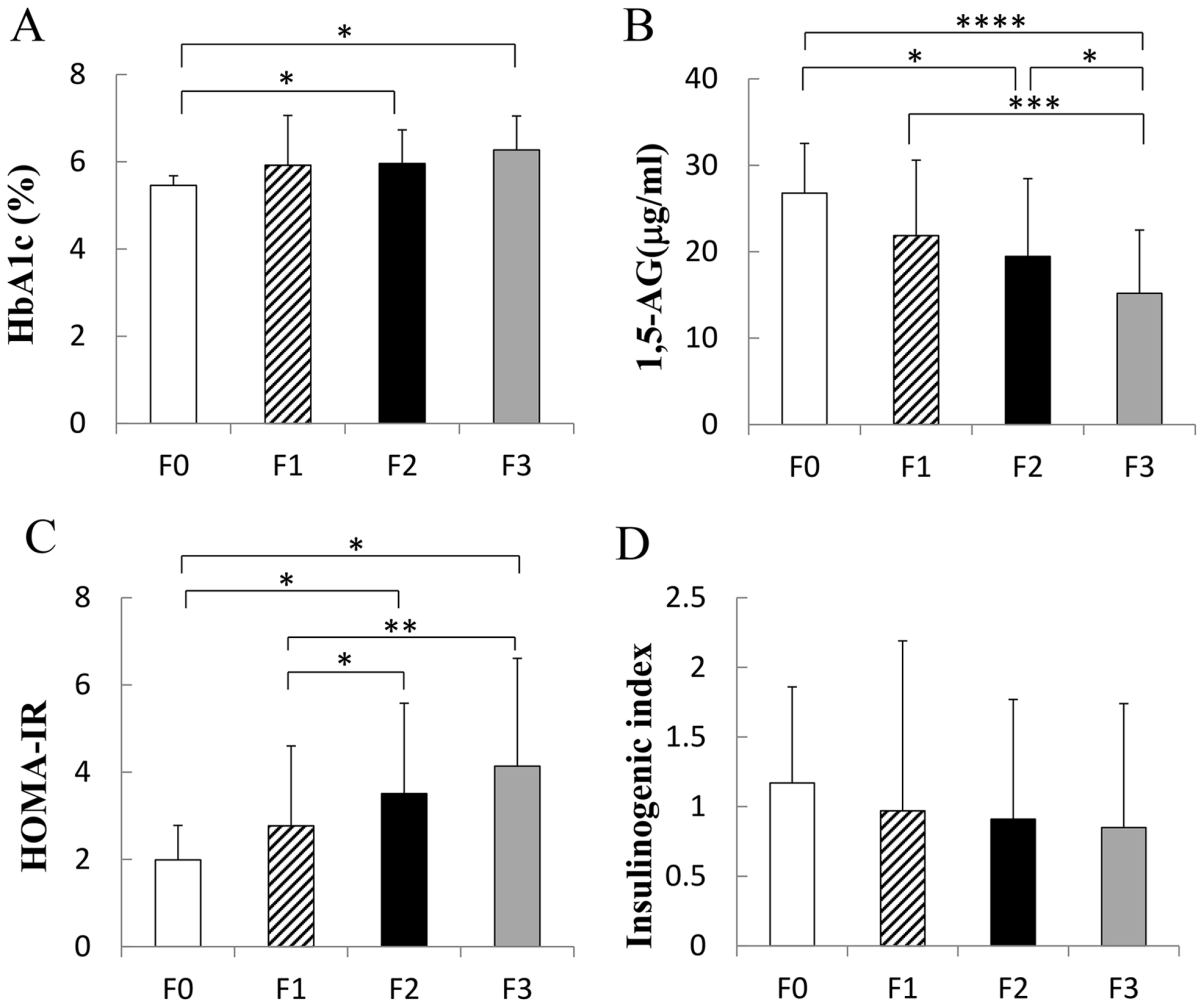
Relationship between hepatic fibrosis and various parameters of glucose metabolism. A) HbA1c was significantly elevated in accordance with the progression of hepatic fibrosis (F0 versus F2, *P<0.05; F0 versus F3, **P<0.05; N = 169). B) 1,5-Anhydroglucitol (1,5-AG) levels were remarkably decreased with the progression of hepatic fibrosis (F0 versus F2, *P<0.05; F0 versus F3, ****P<0.0001; F1 versus F3, ***P<0.001; F2 versus F3, *P<0.05; N = 169). C) HOMA-IR was significantly elevated in the patients with advanced hepatic fibrosis (F0 versus F2, *P<0.05; F0 versus F3, *P<0.05; F1 versus F2, *P<0.05; F1 versus F3, **P<0.01; N = 169). D) The insulinogenic index did not differ among the fibrosis groups (N = 169).

Figure 2A shows that the patients with advanced hepatic fibrosis showed significantly higher levels of HbA1c (F0 versus F2, *P*<0.05; F0 versus F3, *P*<0.05). In addition, 1,5-AG was significantly decreased in accordance with the progression of hepatic fibrosis (F0 versus F2, *P*<0.05; F0 versus F3, *P*<0.0001; F1 versus F3, *P*<0.001; F2 versus F3, *P*<0.05) (Fig. 2B). Severe variability of plasma glucose levels might involve the progression of hepatic fibrosis. HOMA-IR was also elevated in the patients with advanced hepatic fibrosis (F0 versus F2, *P*<0.05; F0 versus F3, *P*<0.05; F1 versus F2, *P*<0.05; F1 versus F3, *P*<0.01) (Fig. 2C). On the other hand, although the insulinogenic index tended to decrease in accordance with the progression of hepatic fibrosis, no statistically significant difference was recognized in our study (Fig. 2D).

**Figure 3:**
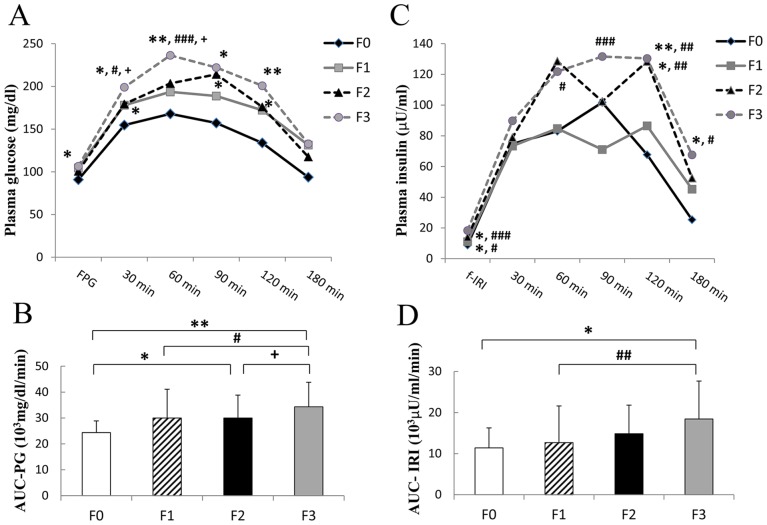
Patterns of glucose and insulin secretion in the 75gOGTT in relation to the progression of hepatic fibrosis. A) The glucose levels were significantly elevated in accordance with the progression of fibrosis (Versus F0: *P<0.05, **P<0.01; Versus F1: ^#^ *P*<0.05, ^###^ *P*<0.001; Versus F2: ^+^ *P*<0.05). B) Area under the curve (AUC) of plasma glucose levels (AUC-PG) was remarkably larger in accordance with the progression of hepatic fibrosis (F0 versus F2, *P<0.05; F0 versus F3, **P<0.01; F1 versus F3, ^#^ *P*<0.05; F2 versus F3, ^+^ *P*<0.05) C) Insulin secretion levels were remarkably higher in the patients with progression of hepatic fibrosis (Versus F0: *P<0.05, **P<0.01; Versus F1: ^#^ *P*<0.05, ^##^ *P*<0.01, ^###^ *P*<0.001). D) AUC of insulin secretion (AUC-IRI) was also significantly larger in accordance with the progression of hepatic fibrosis (F0 versus F3, *P<0.05; F1 versus F3, ^##^ *P*<0.01). (Black diamond: F0: n = 10. Gray square: F1: n = 74. Black triangle: F2: n = 48. Gray circle: F3: n = 37; total, N = 169).

We next evaluated the patterns of glucose and insulin secretion by the 75gOGTT in patients with NAFLD. As shown in Figure 3A, not only the fasting glucose levels (F0 versus F3, *P*<0.05), but also the glucose levels after oral glucose loading (at 30, 60, 90, and 120 minutes) were significantly increased in parallel with the progression of fibrosis. The area under the curve (AUC) of the plasma glucose level (AUC-PG) as the marker for total glucose secretion after oral glucose loading also increased in accordance with the progression of hepatic fibrosis (F0 versus F2, *P*<0.05; F0 versus F3, *P*<0.01; F1 versus F3, *P*<0.05; F2 versus F3, *P*<0.05) (Fig. 3B). In addition, f-IRI was significantly elevated in accordance with the progression of hepatic fibrosis (F0 versus F2, *P*<0.05; F0 versus F3, *P*<0.05; F1 versus F2, *P*<0.05; F1 versus F3, *P*<0.001) (Fig. 3C). Furthermore, the AUC of IRI secretion (AUC-IRI) was also significantly increased in accordance with the progression of hepatic fibrosis (F0 versus F3, *P*<0.05; F1 versus F3, *P*<0.01) (Fig. 3D). In particular, the insulin levels at 120 minutes were remarkably higher in the patients with advanced hepatic fibrosis (F0 versus F2, *P*<0.05; F0 versus F3, *P*<0.01; F1 versus F2, *P*<0.01; F1 versus F3, *P*<0.01). On the other hand, insulin secretion levels at 30 minutes were not statistically different among the groups.

To clarify the prognostic factors associated with advanced hepatic fibrosis, the factors that might be related to glucose metabolism were compared between the mild fibrosis group (F0–2) and severe fibrosis group (F3). Table 2 shows that age, body mass index, HbA1c, f-IRI, HOMA-IR, AUC-IRI, and AUC-PG were significantly higher in the F3 group than in the F0–2 group. Furthermore, 1,5-AG was significantly lower in the F3 group than in the F0–2 group (*P* = 0.00014). In contrast, fasting plasma glucose and the insulinogenic index were not significantly different between these groups. As determined by multivariate logistic regression analysis, 1,5-AG (*P* = 0.008; Z value, -2.65; odds ratio [OR], 0.89509; 95% confidence interval [CI], 0.82473–0.97145) was the only independent factor for association of advanced hepatic fibrosis in patients with NAFLD (Table 3).

**Table 2:** Comparison of the parameters of glucose metabolism between patients with mild fibrosis (F0–2) and severe fibrosis (F3).

	F0-2			F3			P value
	(n = 132)			(n = 37)			
Gender (F/M)	47/85			21/16			
Age (yo)	46.4	±	14.9	53.6	±	14.3	0.00967
BMI (kg/m^2^)	27.4	±	3.0	30.1	±	4.6	0.01395
FPG (mg/dl)	102.1	±	20.7	106.5	±	20.9	0.25665
HbA1c (%)	5.90	±	0.97	6.27	±	0.98	0.04520
f-IRI (μU/ml)	12.0	±	7.5	18.3	±	11.8	0.00012
HOMA-IR	2.98	±	1.92	4.14	±	2.47	0.00320
insulinogenic index	0.96	±	1.06	0.85	±	0.89	0.54574
AUC-IRI (10^3^μU/ml/min)	12.9	±	8.3	18.4	±	9.2	0.00103
AUC-PG (10^3^mg/dl/min)	29.6	±	10.0	34.3	±	9.5	0.01093
1,5-AG (μg/ml)	21.4	±	8.8	15.2	±	7.3	0.00014

P-values were calculated using the Mann–Whitney U test. Data are expressed as mean ± standard deviation. BMI, body mass index; FPG, fasting plasma glucose; f-IRI, fasting immunoreactive insulin; HOMA-IR, homeostasis model assessment of insulin resistance; AUC-IRI, area under the curve of IRI secretion; AUC-PG, area under the curve of plasma glucose; 1,5-AG, 1,5-anhydroglucitol.

**Table 3:** Factors associated with progression of hepatic fibrosis in multivariate logistic regression analysis.

	Odds ratio	95% CI	Z value	P value
Age (yo)	1.04252	1.00009–1.08676	1.96	0.051
BMI (kg/m^2^)	1.08810	0.96010–1.23318	1.32	0.186
HbA1c (%)	0.82385	0.37798–1.79568	−0.49	0.626
f-IRI (μU/ml)	1.15005	0.89106–1.48432	1.07	0.283
HOMA-IR	0.81485	0.32819–2.02311	−0.44	0.659
insulinogenic index	0.59433	0.29955–1.17917	−1.49	0.137
AUC-IRI (10^3^μU/ml/min)	1.00006	0.99997–1.00014	1.29	0.196
AUC-PG (10^3^mg/dl/min)	1.00000	0.99989–1.00011	−0.08	0.937
1,5-AG (μg/ml)	0.89509	0.82473–0.97145	−2.65	0.008

P-values were calculated using logistic regression. BMI, body mass index; FPG, fasting plasma glucose; f-IRI, fasting immunoreactive insulin; HOMA-IR, homeostasis model assessment of insulin resistance; AUC-IRI: area under the curve of IRI secretion; AUC-PG, area under the curve of plasma glucose; 1,5-AG, 1,5-anhydroglucitol; CI, confidence interval.

### Continuous glucose monitoring system (CGMS) clarified that variability of glucose changes was associated with advanced hepatic fibrosis

In multivariate logistic regression analysis, 1,5-AG was selected as the independent associated factor for advanced hepatic fibrosis. A lower 1,5-AG might indicate not only poor control of plasma glucose, but also severe variability of plasma glucose changes. We hypothesized that severe variability of plasma glucose levels might involve the progression of hepatic fibrosis. To address our hypothesis, we investigated the variability of glucose levels during 24 hours by a CGMS. We used the CGMS for 10 patients in the severe fibrosis group (F3–4), including patients with LC, and 10 patients in the mild hepatic fibrosis group (F0–2). No patients in either group took any anti-diabetes drugs or insulin injections. The clinical data of both groups are shown in Table S1 in File S1.

The average median glucose level of the patients with mild fibrosis (F0–2) was significantly lower than that in the patients with severe fibrosis (F3–4) (108.1±12.1 versus 132.8±39.5 mg/dl, *P*<0.00001) (Fig. 4 and Table 4). The variability of median glucose levels of the patients with mild fibrosis was remarkably smaller than that in the patients with severe fibrosis, as shown in Figure 4. The standard deviation of the median glucose levels in the patients with mild fibrosis was remarkably smaller than that in the patients with severe fibrosis (17.4±5.2 versus 39.7±17.8 mg/dl, *P* = 0.0022). In addition, ΔMin–max blood glucose was also significantly larger in patients with severe fibrosis than in those with mild fibrosis (165.0±69.6 versus 115.2±22.8 mg/dl, *P* = 0.0029). Furthermore, all postprandial glucose levels (shadowed areas in Fig. 4, from *P*<0.05 to *P*<0.001) and maximum glucose levels (*P* = 0.0019) (Table 4) in the patients with severe fibrosis were significantly higher than those in the patients with mild fibrosis, although the minimum blood glucose levels were not significantly different (*P* = 0.9221).

**Table 4:** Comparison of variable parameters of continuous glucose monitoring between patients with mild fibrosis (F0–2) and severe fibrosis (F3–4).

Variable	Mild fibrosis (F0-2)			Severe fibrosis (F3-4)			P value
Average median blood glucose (mg/dl)	108.1	±	12.2	131.5	±	34.3	<0.00001
Average standard deviation (mg/dl)	17.4	±	5.2	39.7	±	17.8	0.0022
Minimum blood glucose (mg/dl)	81.7	±	28.7	72.5	±	26.4	0.9221
Maximum blood glucose (mg/dl)	118.8	±	12.5	237.5	±	65.1	0.0019
ΔMin–max blood glucose (mg/dl)	115.2	±	22.8	165.0	±	69.6	0.0029

Average median blood glucose: average median glucose of the patients during the 24-hour monitoring period.

Average standard deviation: average standard deviation of blood glucose of the patients during the 24-hour monitoring period.

Minimum and maximum blood glucose values: lowest and highest values, respectively, during the 24-hour monitoring period.

ΔMin–max blood glucose: difference between minimum and maximum blood glucose. Data are expressed as median ± standard deviation.

**Figure:**
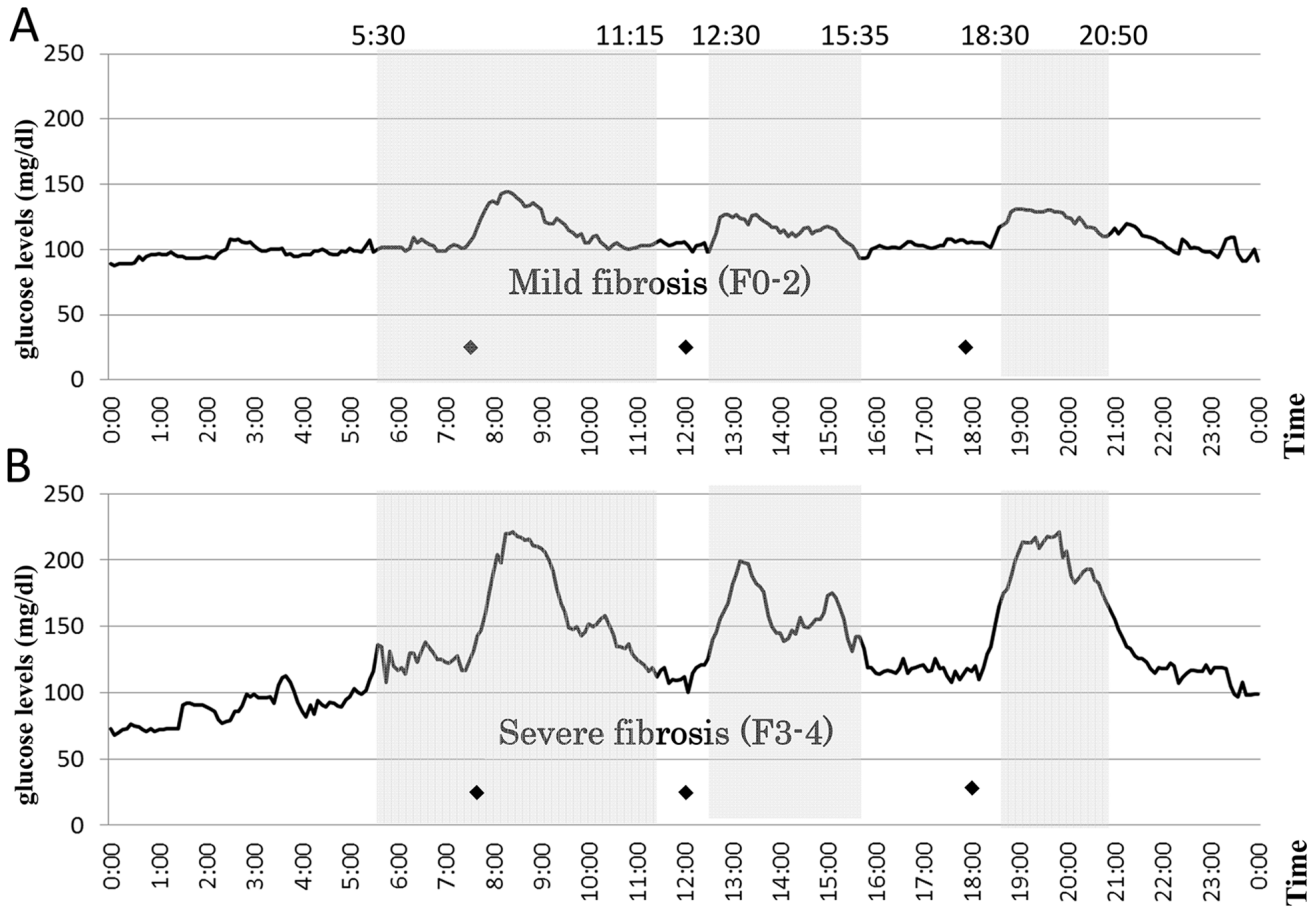
Twenty-four-hour sensor glucose profiles by continuous glucose monitoring system. The changes in the median sensor glucose levels during 24) mild fibrosis (F0–2, *n* = 10) and B) severe fibrosis (F3–4, *n* = 10). The variability of median glucose levels among the patients with mild fibrosis was remarkably smaller than that among the patients with severe fibrosis. Median glucose levels in the patients with severe fibrosis were higher than those with mild fibrosis (shadows areas, P<0.05 to P<0.001). Black diamond: Time of meal consumption.

## Discussion

The number of patients with NAFLD and NASH has increased according to the increase in the prevalence of patients with obesity and T2DM worldwide. Patients with NAFLD and NASH often have metabolic disorders including IR and T2DM. In particular, IR is considered to be one of most important background factors for the development of NAFLD and NASH. However, detailed clinical features of impairment of glucose metabolism in patients with NAFLD and NASH are not well understood. In this study, we clarified the predictive factors in glucose metabolism for the development of hepatic fibrosis in patients with NAFLD by the 75gOGTT and CGMS methods.

We evaluated the relationship between the prevalence of T2DM diagnosed by the 75gOGTT and the degree of hepatic fibrosis in patients with NAFLD (Fig. 1). No patients with F0 fibrosis had T2DM, but 80% had NGT. On the other hand, the prevalence of NGT in the patients with F3 fibrosis was only 21.6%, and the prevalence of T2DM was 48.6%. In accordance with the progression of hepatic fibrosis, the prevalence of patients with T2DM was significantly increased and that of NGT was significantly decreased. T2DM is reportedly an independent predictor for the progression of hepatic fibrosis in patients with NAFLD [10]. Our data also indicated that the development of T2DM might induce the development of hepatic fibrosis in patients with NAFLD. On the other hand, however, the presence of NASH and NAFLD themselves is reportedly associated with a high risk of developing T2DM [12].

According to the clinical and physiological data of the four groups of patients with NAFLD (Table 1), age, aspartate aminotransferase, hepatic fibrosis markers, and ferritin were higher and platelets were lower in accordance with the progression of hepatic fibrosis, as previously reported [8], [23]. To clarify the detailed glucose impairment related to the progression of hepatic fibrosis, we evaluated the various parameters of glucose metabolism. HbA1c was gradually elevated with the progression of hepatic fibrosis (Fig. 2A). Although HbA1c in the patients with F3 fibrosis was higher than that in the patients with F0 fibrosis, HbA1c in all fibrosis groups was around 6.0% because the glucose impairment in all patients in this study was mild enough to perform the 75gOGTT. On the other hand, 1,5-AG remarkably gradually decreased in accordance with the progression of hepatic fibrosis (Fig. 2B). Considering both the results of HbA1c and those of 1,5-AG, evaluation of 1,5-AG might more closely reflect the glycemic variability in patients with NAFLD, and glycemic variability would be closely related to the progression of hepatic fibrosis. IR is considered to be one of the most important predictive factors for the development of NAFLD and NASH [11]. Therefore, evaluation of HOMA-IR also was investigated in our study. HOMA-IR also gradually increased in accordance with the progression of hepatic fibrosis (Fig. 2C). However, the insulinogenic index, the ability of early insulin secretion, was not significantly different among the groups (Fig. 2D).

We next investigated and evaluated the clinical features of 75gOGTT in patients with NAFLD in relation to the progression of hepatic fibrosis (Fig. 3). After oral glucose loading, glucose levels were increased in the patients with advanced fibrosis (F3) compared with the patients with mild fibrosis (F0–2) (F3 versus F0, *P*<0.01; F3 versus F1, *P*<0.001; F3 versus F2, *P*<0.05 at 60 minutes) (Fig. 3A). The elevation of glucose levels continued until 120 minutes after oral glucose loading in the patients with F3 fibrosis. In addition, the AUC–PG as the marker for total glucose secretion after oral glucose loading also significantly gradually increased in accordance with the progression of hepatic fibrosis (F0 versus F2, *P*<0.05; F0 versus F3, *P*<0.01; F1 versus F, *P*<0.05; F2 versus F3, *P*<0.05) (Fig. 3B). Furthermore, f-IRI was significantly elevated in accordance with the progression of hepatic fibrosis (F0 versus F2, *P*<0.05; F0 versus F3, *P*<0.05; F1 versus F2, *P*<0.05; F1 versus F3, *P*<0.001) (Fig. 3C). These results are agreement with the results in Figure 2C. After oral glucose loading, insulin secretion was relatively quickly elevated in all groups of patients with NAFLD. Insulin secretion levels at 30 minutes were not statistically different among the groups. This result is in agreement with the results of the insulinogenic index (Fig. 2D). The insulin secretion in the mild fibrosis group (F0 and F1) decreased relatively early with the decrease in blood glucose levels. On the other hand, insulin secretion in the advanced fibrosis groups continued until 120 minutes. Therefore, the insulin levels at 120 minutes were remarkably higher in the patients with advanced hepatic fibrosis (F0 versus F2, *P*<0.05; F0 versus F3, *P*<0.01; F1 versus F2, *P*<0.01; F1 versus F3, *P*<0.01), as previously reported [24], [25]. Furthermore, the AUC-IRI was also significantly increased in accordance with the progression of hepatic fibrosis (F0 versus F3, *P*<0.05; F1 versus F3, *P*<0.01) (Fig. 3D). It is known that insulin has the potential to function as a growth factor. IR and/or T2DM reportedly may accelerate the progression of NASH through lipogenesis, inflammation, and fibrogenesis [26] and induce cancer growth [27]. Kaji et al. also reported that not only glucose and insulin alone, but also a combination of the two, stimulated the proliferation and activation of hepatic stellate cells. They concluded that the IR status directly accelerates the development of hepatic fibrosis and hepatocarcinogenesis through activation of hepatic stellate cells [28]. Taken together with our results, hyperinsulinemia and hyperglycemia might be related to the progression of hepatic fibrosis in NAFLD.

To elucidate the predictive factors that are associated with the development of hepatic fibrosis in NAFLD, various parameters of glucose metabolism were compared between the mild fibrosis group (F0–2) and severe fibrosis group (F3). In univariate analysis (Table 2), age (*P* = 0.00967), body mass index (*P* = 0.01395), HbA1c (*P* = 0.0452), f-IRI (*P* = 0.00012), HOMA-IR (*P* = 0.0032), AUC-IRI (*P* = 0.00103), and AUC-PG (*P* = 0.01093) were significantly higher and 1,5-AG (*P* = 0.00014) was significantly lower in the severe fibrosis group than in the mild fibrosis group (Table 1, Figs. 2 and 3). As determined by multivariate logistic regression analysis, only 1,5-AG (*P* = 0.008; Z value, −2.65; OR, 0.89509; 95% CI, 0.82473–0.97145) remained as the independently associated factor for advanced fibrosis (Table 3). As mentioned in the above results, it was considered that 1,5-AG might have reflected the glycemic variability in patients with NAFLD in this study.

To confirm the relationship between glycemic variability and progression of hepatic fibrosis in NAFLD, the changes in blood glucose levels during 24 hours were monitored by the CGMS in the patients with NAFLD with severe fibrosis (F3–4, *n* = 10) and mild fibrosis (F0–2, *n* = 10). CGMS examinations were performed at the inpatient center of Kochi Medical School, and the timing of meals and calories contained in the meals were strictly standardized during the examination. In this study, the severe fibrosis group (F3–4, *n* = 10) included three patients with LC (F4) who were all diagnosed with T2DM and whose hyperglycemia was too high to perform the 75gOGTT. However, none of the patients in this study took any anti-diabetic drugs or insulin injections. Figure 4 shows that the variability of the median glucose levels of the patients with mild fibrosis (F0–2) was remarkably smaller than that of the patients with severe fibrosis (F3–4). Furthermore, the standard deviation in severe fibrosis (39.7±17.8 mg/dl) was much larger than that in mild fibrosis (17.4±5.2 mg/dl, *P* = 0.0022) (Table 4). Although the minimum blood glucose levels in patients with severe fibrosis tended to be lower than those in patients with mild fibrosis (72.5±26.4 versus 81.7±28.7 mg/dl, *P* = 0.9221), the maximum blood glucose level in patients with severe fibrosis was remarkably higher than that in patients with mild fibrosis (237.5±65.1versus 118.8±12.5 mg/dl, *P* = 0.0019) (Table 4). As a result, the ΔMin–max blood glucose was also significantly larger in severe fibrosis than in mild fibrosis (165.0±69.6 versus 115.2±22.8 mg/dl, *P* = 0.0029). The shadowed areas, which indicate glucose levels, were statistically different between the mild and severe fibrosis groups showed postprandial hyperglycemia, and the hyperglycemias were long continued (Fig. 4). Moreover, we noticed a specific clinical feature of postprandial hyperglycemia in patients with NAFLD. The peaks of postprandial hyperglycemia occurred 1 hour after every meal. Interestingly, glucose levels from midnight to early morning tended to be lower in patients with severe fibrosis and had become elevated by breakfast. The statistical differences in these parameters between the mild and severe fibrosis groups did not change even when three patients with LC (F4) were excluded from the severe fibrosis group (Figure S1 and Tables S2 and S3 in File S1). Moreover, in chronic hepatitis C, even in liver cirrhosis, the changes in blood glucose didn't necessarily show any certain patterns unlike NAFLD (data not shown). Taken together with our results, severe variability of blood glucose changes might be closely related to the progression of hepatic fibrosis in NAFLD.

Postprandial hyperglycemia and glycemic variability are reportedly involved in the progression of atherosclerosis through an increase in oxidative stress, activation of inflammatory cytokines and inflammation [13]–[15], and induction of other pathogenic complications [29], [30], [31]. In addition, repetitive postprandial glucose fluctuation reportedly evokes more pronounced adhesion of monocytes to endothelial cells compared with that induced by stable hyperglycemia [32]. In addition, the main mechanism of monocyte adhesion to endothelial cells has been shown to be increased serum adrenaline induced by postprandial glucose spikes [33]. Furthermore, the importance of glucose variability was recently recognized as an independent factor associated with increasing mortality in patients with diabetes [34], [35] and critically ill patients [36], [37]. Oxidative stress is well known as one of most important factors for inflammation and progression of hepatic fibrosis in NAFLD [16], [17]. Taken together with our results, therefore, variability of blood glucose might also induce monocyte adhesion to endothelial cells, activate inflammatory cytokines and inflammation, and increase oxidative stress in the liver of patients with NAFLD.

There are several limitations of this study. We showed that the prevalence of patients with T2DM was significantly increased (Fig. 1) and age and BMI tended to increase (Table 1) in accordance with the progression of hepatic fibrosis. It is known that age and BMI contribute to both the prevalence of T2DM and the progression of hepatic fibrosis. Therefore, not only progression of T2DM, but also age and BMI, might have influenced the progression of hepatic fibrosis in this study.

In conclusion, we clarified that hyperinsulinemia and hyperglycemia are important predictive factors for the development of hepatic fibrosis in this study. More importantly, variability of blood glucose is one of most important predictive factors in glucose impairment for progression of hepatic fibrosis in NAFLD. Therefore, we might need to reconsider the use of anti-diabetic drugs to inhibit the progression of hepatic fibrosis during treatment of patients with NAFLD.

## Supporting Information

File S1 **Supplemental Figures and Tables. Figure S1, Twenty-four-hour sensor glucose profiles by continuous glucose monitoring system.** The changes in the median sensor glucose levels during 24 hours are shown in the patients with A) F0–2 fibrosis (*n* = 10) and B) F3 fibrosis (*n* = 7). The variability of median glucose levels among the patients with F0–2 fibrosis was remarkably smaller than that among the patients with F3 fibrosis. Median glucose levels in the patients with F3 fibrosis were higher than those with F0–2 fibrosis (shadows areas, P<0.05 to P<0.001): Time of meal consumption. **Table S1, Comparison of the clinical and physiological characteristics between the patients with mild fibrosis (F0–2) and severe fibrosis (F3–4).** Data are expressed as median ± standard deviation. BMI, body mass index; AST, aspartate aminotransferase; ALT, alanine aminotransferase; GGT, gamma-glutamyl transpeptidase; ChE, cholinesterase; T-Cho, total cholesterol; TG, triglycerides; FPG, fasting plasma glucose; Plt, platelets; Fe, plasma iron; HA, hyaluronic acid; IV collagen 7S, type IV collagen 7S; P-3-P, type III procollagen N-peptide. **Table S2, Comparison of the clinical and physiological characteristics between the patients with F0–2 fibrosis and F3 fibrosis.** Data are expressed as median ± standard deviation. BMI, body mass index; AST, aspartate aminotransferase; ALT, alanine aminotransferase; GGT, gamma-glutamyl transpeptidase; ChE, cholinesterase; T-Cho, total cholesterol; TG, triglycerides; FPG, fasting plasma glucose; Plt, platelets; Fe, plasma iron; HA, hyaluronic acid; IV collagen 7S, type IV collagen 7S; P-3-P, type III procollagen N-peptide. **Table S3, Comparison of variable parameters of continuous glucose monitoring between patients with F0–2fibrosis and F3 fibrosis.** Average median blood glucose: average median glucose of the patients during the 24-hour monitoring period. Average standard deviation: average standard deviation of blood glucose of the patients during the 24-hour monitoring period. Minimum and maximum blood glucose values: lowest and highest values, respectively, during the 24-hour monitoring period ΔMin–max blood glucose: difference between minimum and maximum blood glucose. Data are expressed as median ± standard deviation.
(DOCX)
