# Supplementary material for: Glycemic Variability Is an Independent Predictive Factor for Development of Hepatic Fibrosis in Nonalcoholic Fatty Liver Disease
Source: PLoS One. 2013 Nov 6;8(11):e76161. doi: 10.1371/journal.pone.0076161 (PMC3819352; doi:10.1371/journal.pone.0076161)
Supplement: File S1 — Supplemental Figures and Tables. Figure S1, Twenty-four-hour sensor glucose profiles by continuous glucose monitoring system. The changes in the median sensor glucose levels during 24 hours are shown in the patients with A) F0–2 fibrosis (n = 10) and B) F3 fibrosis (n = 7). The variability of median glucose levels among the patients with F0–2 fibrosis was remarkably smaller than that among the patients with F3 fibrosis. Median glucose levels in the patients with F3 fibrosis were higher than those with F0–2 fibrosis (shadows areas, P<0.05 to P<0.001): Time of meal consumption. Table S1, Comparison of the clinical and physiological characteristics between the patients with mild fibrosis (F0–2) and severe fibrosis (F3–4). Data are expressed as median ± standard deviation. BMI, body mass index; AST, aspartate aminotransferase; ALT, alanine aminotransferase; GGT, gamma-glutamyl transpeptidase; ChE, cholinesterase; T-Cho, total cholesterol; TG, triglycerides; FPG, fasting plasma glucose; Plt, platelets; Fe, plasma iron; HA, hyaluronic acid; IV collagen 7S, type IV collagen 7S; P-3-P, type III procollagen N-peptide. Table S2, Comparison of the clinical and physiological characteristics between the patients with F0–2 fibrosis and F3 fibrosis. Data are expressed as median ± standard deviation. BMI, body mass index; AST, aspartate aminotransferase; ALT, alanine aminotransferase; GGT, gamma-glutamyl transpeptidase; ChE, cholinesterase; T-Cho, total cholesterol; TG, triglycerides; FPG, fasting plasma glucose; Plt, platelets; Fe, plasma iron; HA, hyaluronic acid; IV collagen 7S, type IV collagen 7S; P-3-P, type III procollagen N-peptide. Table S3, Comparison of variable parameters of continuous glucose monitoring between patients with F0–2fibrosis and F3 fibrosis. Average median blood glucose: average median glucose of the patients during the 24-hour monitoring period. Average standard deviation: average standard deviation of blood glucose of the patients during the 24-hour moni [file pone.0076161.s001.docx]

Table S1. Comparison of the clinical and physiological characteristics between the patients with mild fibrosis (F0–2) and severe fibrosis (F3–4)

|  | F0-2 (n=10) | | | F3-4 (n=10) | | | P value |
| --- | --- | --- | --- | --- | --- | --- | --- |
| Gender (F/M) | 3/7 | | | 5/5 | | |  |
| Fibrosis stage (F0/F1/F2/F3/F4) | 1 / 5 / 4 / - / - | | | - / - / - / 7/ 3 | | |  |
| DM / IGT・IFG / NGT | 1 / 2 / 7 | | | 7 / 2 / 1 | | |  |
| Age (yo) | 43.7 | ± | 20.9 | 61.2 | ± | 10.0 | < 0.05 |
| BMI (kg/m^2^) | 30.9 | ± | 6.5 | 29.6 | ± | 11.5 | ns |
| AST (IU/L) | 54.0 | ± | 26.0 | 69.1 | ± | 23.7 | ns |
| ALT (IU/L) | 80.6 | ± | 34.5 | 70.8 | ± | 36.0 | ns |
| ALP (IU/L) | 250.4 | ± | 44.9 | 301.3 | ± | 67.8 | ns |
| GGT (IU/L) | 66.3 | ± | 47.1 | 132.3 | ± | 93.0 | ns |
| ChE (IU/L) | 381.7 | ± | 105.6 | 298.9 | ± | 86.1 | ns |
| Albumin (g/dL) | 4.74 | ± | 0.37 | 4.30 | ± | 0.42 | < 0.05 |
| BUN (mg/dL) | 16.2 | ± | 3.8 | 13.5 | ± | 4.5 | ns |
| Crn (mg/dL) | 0.78 | ± | 0.15 | 0.61 | ± | 0.20 | ns |
| FPG (mg/dl) | 103.0 | ± | 15.7 | 120.8 | ± | 34.6 | ns |
| HbA1c (%) | 5.71 | ± | 0.77 | 7.11 | ± | 1.34 | < 0.05 |
| 1,5-AG (μg/ml) | 19.6 | ± | 6.8 | 10.2 | ± | 5.5 | < 0.01 |
| TC (mg/dL) | 203.9 | ± | 33.5 | 198.5 | ± | 33.4 | ns |
| TG (mg/dL) | 168.4 | ± | 89.5 | 151.3 | ± | 87.2 | ns |
| RBC (x 10^4^/ml) | 489.6 | ± | 50.3 | 432.9 | ± | 51.0 | < 0.05 |
| Hb (g/dL) | 15.3 | ± | 1.4 | 13.8 | ± | 1.29 | < 0.05 |
| Plt (x 10^4^/ml) | 22.9 | ± | 6.9 | 13.5 | ± | 5.9 | < 0.01 |
| WBC (x 10^3^/ml) | 6.03 | ± | 1.60 | 5.71 | ± | 2.14 | ns |
| Fe (mg/dL) | 130.0 | ± | 42.2 | 135.0 | ± | 50.6 | ns |
| Ferritin (ng/ml) | 222.1 | ± | 86.3 | 295.0 | ± | 282.8 | ns |
| HA (ng/ml) | 50.2 | ± | 71.8 | 308.1 | ± | 206.1 | < 0.01 |
| IVcollagen7S (ng/ml) | 3.33 | ± | 1.39 | 6.43 | ± | 1.99 | < 0.01 |
| P-3-P (U/ml) | 1.02 | ± | 1.42 | 0.95 | ± | 0.30 | ns |

Data are expressed as median ± standard deviation. BMI, body mass index; AST, aspartate aminotransferase; ALT, alanine aminotransferase; GGT, gamma-glutamyl transpeptidase; ChE, cholinesterase; T-Cho, total cholesterol; TG, triglycerides; FPG, fasting plasma glucose; Plt, platelets; Fe, plasma iron; HA, hyaluronic acid; IV collagen 7S, type IV collagen 7S; P-3-P, type III procollagen N-peptide

Table S2. Comparison of the clinical and physiological characteristics between the patients with F0–2 fibrosis and F3 fibrosis

|  | F0-2 (n=10) | | | F3 (n=7) | | | P value |
| --- | --- | --- | --- | --- | --- | --- | --- |
| Gender (F/M) | 3/7 | | | 4/3 | | |  |
| Fibrosis stage (F0/F1/F2/F3/F4) | 1 / 5 / 4 / - / - | | | - / - / - / 7 / - | | |  |
| DM / IGT・IFG / NGT | 1 / 2 / 7 | | | 5 / 2 / 0 | | |  |
| Age (yo) | 43.7 | ± | 20.9 | 62.1 | ± | 7.8 | < 0.05 |
| BMI (kg/m^2^) | 30.9 | ± | 6.5 | 26.9 | ± | 4.99 | ns |
| AST (IU/L) | 54.0 | ± | 26.0 | 76.1 | ± | 24.1 | ns |
| ALT (IU/L) | 80.6 | ± | 34.5 | 84.1 | ± | 33.8 | ns |
| ALP (IU/L) | 250.4 | ± | 44.9 | 304.6 | ± | 71.7 | ns |
| GGT (IU/L) | 66.3 | ± | 47.1 | 123.9 | ± | 100.7 | ns |
| ChE (IU/L) | 381.7 | ± | 105.6 | 328.4 | ± | 73.4 | ns |
| Albumin (g/dL) | 4.74 | ± | 0.37 | 4.43 | ± | 0.21 | ns |
| BUN (mg/dL) | 16.2 | ± | 3.8 | 14.8 | ± | 3.3 | ns |
| Crn (mg/dL) | 0.78 | ± | 0.15 | 0.61 | ± | 0.22 | ns |
| FPG (mg/dl) | 103.0 | ± | 15.7 | 122.7 | ± | 41.0 | ns |
| HbA1c (%) | 5.71 | ± | 0.77 | 7.46 | ± | 1.28 | < 0.01 |
| 1,5-AG (μg/ml) | 19.6 | ± | 6.8 | 10.4 | ± | 6.4 | < 0.05 |
| TC (mg/dL) | 203.9 | ± | 33.5 | 209.4 | ± | 32.4 | ns |
| TG (mg/dL) | 168.4 | ± | 89.5 | 165.6 | ± | 111.0 | ns |
| RBC (x 10^4^/ml) | 489.6 | ± | 50.3 | 419.6 | ± | 49.7 | < 0.05 |
| Hb (g/dL) | 15.3 | ± | 1.4 | 13.4 | ± | 1.1 | < 0.01 |
| Plt (x 10^4^/ml) | 22.9 | ± | 6.9 | 15.7 | ± | 5.4 | < 0.05 |
| WBC (x 10^3^/ml) | 6.03 | ± | 1.60 | 6.01 | ± | 2.48 | ns |
| Fe (mg/dL) | 130.0 | ± | 42.2 | 122.4 | ± | 54.1 | ns |
| Ferritin (ng/ml) | 222.1 | ± | 86.3 | 347.8 | ± | 282.8 | ns |
| HA (ng/ml) | 50.2 | ± | 71.8 | 308.1 | ± | 308.0 | < 0.01 |
| IVcollagen7S (ng/ml) | 3.33 | ± | 1.39 | 5.70 | ± | 1.11 | < 0.01 |
| P-3-P (U/ml) | 1.02 | ± | 1.42 | 0.80 | ± | 0.12 | ns |

Data are expressed as median ± standard deviation. BMI, body mass index; AST, aspartate aminotransferase; ALT, alanine aminotransferase; GGT, gamma-glutamyl transpeptidase; ChE, cholinesterase; T-Cho, total cholesterol; TG, triglycerides; FPG, fasting plasma glucose; Plt, platelets; Fe, plasma iron; HA, hyaluronic acid; IV collagen 7S, type IV collagen 7S; P-3-P, type III procollagen N-peptide

Table S3. Comparison of variable parameters of continuous glucose monitoring between patients with F0–2fibrosis and F3 fibrosis

| Variable | F0 - F2 fibrosis  (n=1) | | | F3 fibrosis  (n=7) | | | P value |
| --- | --- | --- | --- | --- | --- | --- | --- |
| Average median blood glucose (mg/dl) | 108.1 | ± | 12.2 | 133.7 | ± | 31.6 | <0.00001 |
| Average standard deviation (mg/dl) | 17.4 | ± | 5.2 | 38.6 | ± | 21.1 | 0.00109 |
| Minimum blood glucose (mg/dl) | 81.7 | ± | 28.7 | 71.9 | ± | 27.2 | 0.3499 |
| Maximum blood glucose(mg/dl) | 118.8 | ± | 12.5 | 236.1 | ± | 75.9 | 0.0109 |
| ΔMin–max blood glucose(mg/dl) | 115.2 | ± | 22.8 | 164.3 | ± | 80.8 | 0.0063 |

Average median blood glucose: average median glucose of the patients during the 24-hour monitoring period

Average standard deviation: average standard deviation of blood glucose of the patients during the 24-hour monitoring period

Minimum and maximum blood glucose values: lowest and highest values, respectively, during the 24-hour monitoring period

ΔMin–max blood glucose: difference between minimum and maximum blood glucose. Data are expressed as median ± standard deviation.

Figure S1


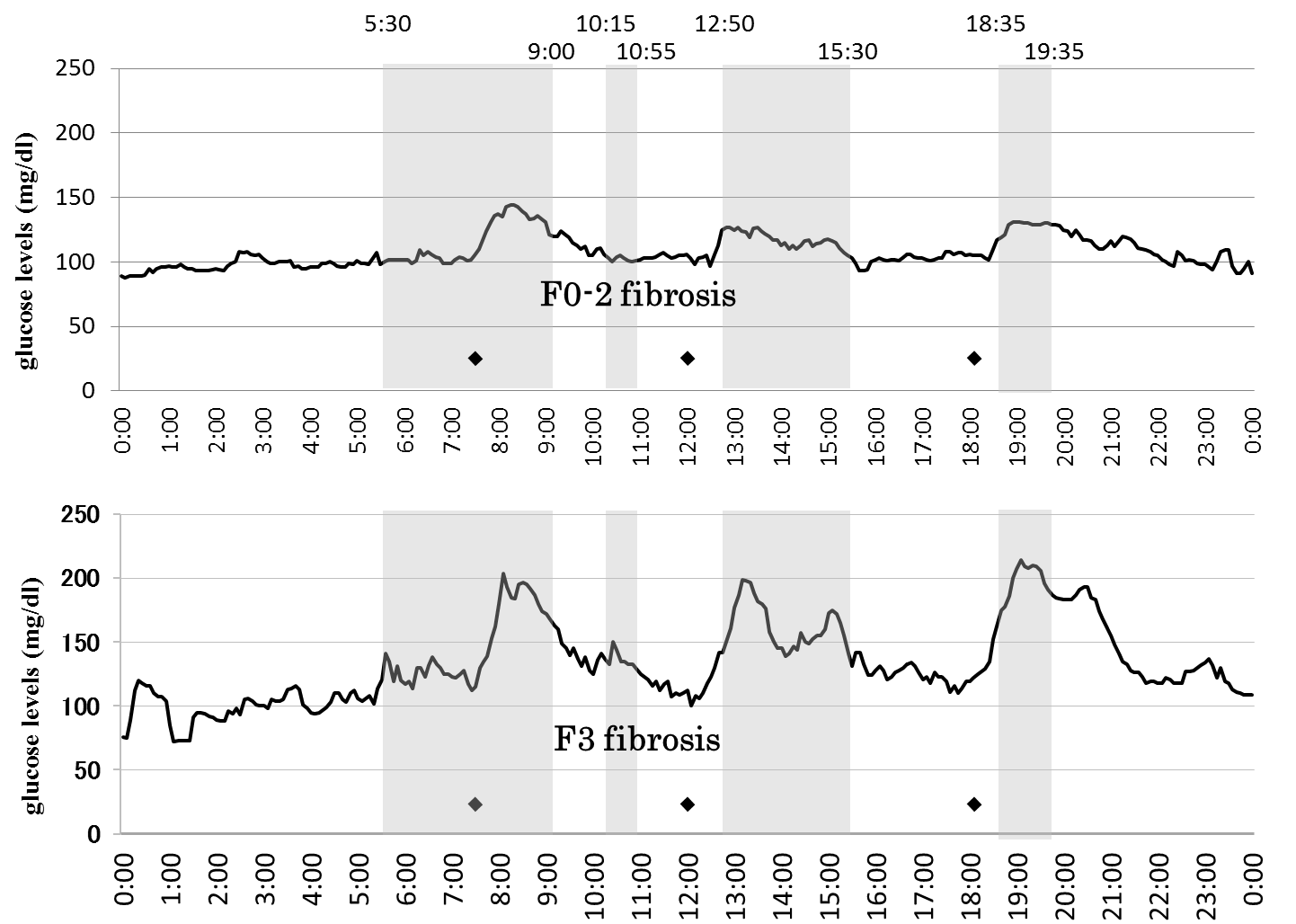


**Figure S1 Twenty-four-hour sensor glucose profiles by continuous glucose monitoring system**

The changes in the median sensor glucose levels during 24 hours are shown in the patients with A) F0–2 fibrosis (*n* = 10) and B) F3 fibrosis (*n* = 7). The variability of median glucose levels among the patients with F0–2 fibrosis was remarkably smaller than that among the patients with F3 fibrosis. Median glucose levels in the patients with F3 fibrosis were higher than those with F0–2 fibrosis (shadows areas, P < 0.05 to P < 0.001). : Time of meal consumption
